# Face Mask: As a Source or Protector of Human Exposure to Microplastics and Phthalate Plasticizers?

**DOI:** 10.3390/toxics11020087

**Published:** 2023-01-17

**Authors:** Jiong Cao, Yumeng Shi, Mengqi Yan, Hongkai Zhu, Shucong Chen, Ke Xu, Lei Wang, Hongwen Sun

**Affiliations:** MOE Key Laboratory of Pollution Processes and Environmental Criteria, College of Environmental Science and Engineering, Nankai University, Tianjin 300350, China

**Keywords:** face mask, microplastics, phthalates, risk assessment, respiratory exposure

## Abstract

Wearing masks has become the norm during the Coronavirus disease pandemic. Masks can reportedly interface with air pollutants and release microplastics and plastic additives such as phthalates. In this study, an experimental device was set up to simulate the impact of five kinds of masks (activated-carbon, N95, surgical, cotton, and fashion masks) on the risk of humans inhaling microplastics and phthalates during wearing. The residual concentrations of seven major phthalates ranged from 296 to 72,049 ng/g (median: 1242 ng/g), with the lowest and the highest concentrations detected in surgical (median: 367 ng/g) and fashion masks (median: 37,386 ng/g), respectively. During the whole inhalation simulation process, fragmented and 20–100 μm microplastics accounted for the largest, with a rapid release during the first six hours. After one day’s wearing, that of 6 h, while wearing different masks, 25–135 and 65–298 microplastics were inhaled indoors and outdoors, respectively. The total estimated daily intake of phthalates with indoor and outdoor conditions by inhalation and skin exposure ranged from 1.2 to 13 and 0.43 to 14 ng/kg bw/d, respectively. Overall, surgical masks yield a protective effect, while cotton and fashion masks increase human exposure to microplastics and phthalates both indoors and outdoors compared to no mask wearing. This study observed possible risks from common facemasks and provided suggestions to consumers for selecting suitable masks to reduce exposure risks from microplastics and phthalate acid.

## 1. Introduction

Since the outbreak of Coronavirus disease (COVID-19), more than 500 million people have been infected, mainly by droplets and aerosol transmission [1,2]. The World Health Organization recommends mask-wearing as one of the best safeguard measures for breaking the transmission chain of the virus during the pandemic [3]. It has been reported that the attributable risk was about six times higher in non-mask wearers than that of mask wearers [4]. Accordingly, mask-wearing has become a norm, with 129 billion masks used every month in 2020 [5]. Although surgical masks, N95 respirators, and similar masks yield a more significant protective effect than other masks [6], they are often costly. As a result, activated carbon masks, cotton masks, and fashion masks have gained significant momentum.

However, the wide use of masks has adversely affected the environment. Disposable masks are made of polymers such as polypropylene, polyurethane, polyacrylonitrile, polystyrene, polycarbonate, polyethylene, or polyester [7]. Many additives, including plasticizers, antioxidants, and flame retardants, are added to these materials in non-covalent forms to obtain products with greater performance [8]. Phthalate esters (PAEs), a common kind of plastic additives, have been detected in facemasks with a level up to 38 μg/g [9]. Used masks undergo oxidation or weathering into microplastics after discarding them into the environment when not properly disposed of, contributing to the ubiquitous occurrence of chemical additives and microplastics in nature [10,11].

Significantly, these emerging contaminants in masks affect the environment and are deleterious to humans during wearing. Once inhaled, part of microplastics keep away from the clearing mechanism of the respiratory tract and may lead to lung disease, such as asthma, chronic obstructive pulmonary disease, and even cancer, by producing radical oxygen species, inducing inflammation, damaging cellular structures, and blocking vessels [12,13]. PAEs, typical endocrine disrupters, can enter the body through dermal contact and inhalation while wearing masks and result in a series of endocrine disorders. Di (2-ethylhexyl) phthalate (DEHP), for example, can decrease female fertility [14], increase risk of allergic diseases and asthma in children [15], cause insulin resistance [16], and be associated with overweight and obesity [17]. Nonetheless, few studies have focused on the two pollutants’ extent of harm to human when wearing masks. Li et al. [18] found that masks released fibrous microplastics after 720 h but did not consider the indoor and outdoor conditions, and the interval time was excessively long, as the average wearing time of masks was less than 24 h. Although the estimated daily intake of PAEs based on the content reduction after degassing the mask has been calculated, about 2.0–20 ng/kg bw/day for adults [19,20], release characteristics of PAEs and various masks’ different effects on exposure were not concerned.

Moreover, it is widely thought that masks represent a protector of human exposure to microplastics and phthalates. Overwhelming evidence substantiates that these pollutants are widely distributed in the air, with a definite risk of inhalation exposure when people do not wear masks [21,22]. Therefore, whether facemasks increase or decrease exposure to microplastics and phthalates remains uncertain. Herein, we detected PAEs in five types of masks, including activated-carbon masks, N95 masks, surgical masks, cotton masks, and fashion masks, and we developed simulated inhalation equipment based on the report of Li et al. [18], which was applied for collecting microplastics and phthalates during 24 h indoors and outdoors to further identify the effects of different facemasks while wearing.

## 2. Materials and Methods

### 2.1. Chemicals and Reagents

Seven phthalate diester standards including dimethyl phthalate (DMP), diethyl phthalate (DEP), di-iso-butyl phthalate (DIBP), di-n-butyl phthalate (DNBP), butyl benzyl phthalate (BBZP), di(2-ethylhexyl) phthalate (DEHP), and di-n-octyl phthalate (DNOP) were purchased from Dr. Ehrenstorfer (Augsburg, Germany; purity ≥ 99.0%). Two isotope-labelled standards, namely d4-DNBP and d4-DEHP, were purchased from Sigma Aldrich (St. Louis, MO, USA; purity ≥ 96.7%). Details with regard to these target chemicals are listed in Table S1 in the Supporting Information (SI). Hexane and methanol used were of high-performance liquid chromatography (HPLC) grade and purchased from Anpel (Shanghai, China). The glass fiber filter (GFF; 0.45-μm pore size, Ø 25 mm) was purchased from Beihua (Beijing, China).

### 2.2. Facemask Collection

A total of 11 brands of best-selling facemasks representing 5 main types (as shown in Figure S1), i.e., activated carbon (hereinafter referred to as “AC”; sample code: M1–2), N95 (M3–5), surgical (SU; M6–7), cotton (CO; M8–9) and fashion masks (FA; M10–11), were purchased from online retailers in October 2021 and then sealed in polyethylene bags for storage at 4 °C in the dark until exposure experiment or chemical analysis. Each category of facemask contained at least two items. All facemask samples were manufactured in China, and the unit price of each facemask varied from 0.12 to 10 CNY. The majority of the facemasks were made of non-woven and melt-blown fabrics except for cotton and fashion masks whose main bodies were cotton and polyurethane, respectively. A detailed list of samples analyzed in this study is provided in Table S2.

### 2.3. Experimental Approach

**Figure 1:**
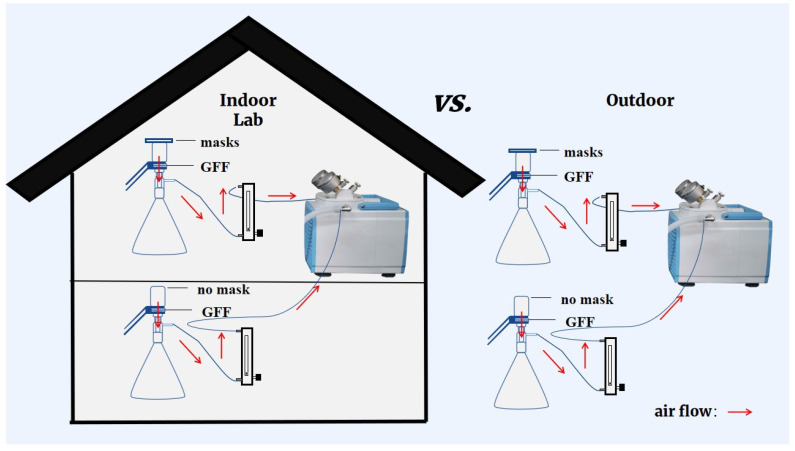
Diagram explaining multiple experimental scenarios for wearing different kinds of masks in indoor or outdoor environments.

The experimental system that had been reported by Li et al. [18] was adopted in the present study with minor modification. As shown in Figure 1, each experimental flow-path was composed of a mask sample, a suction flask with GFF filter, a rotermeter, and a vacuum pump. In order to simulate the human inhalation process, air was sucked through the testing mask continuously at a flow rate of 15 L/min [23]. A blank test collecting microplastics and phthalates without the mask fixed on top of the suction flask was also conducted at the same time to figure out the role facemasks played. This experiment was conducted indoors and outdoors, and no contamination control measures of sampling environments were applied to reflect a realistic situation of microplastics and phthalate diester inhalation (Figure 1).

### 2.4. PAEs Measurements

Samples from several parts of the unused mask (without ear loops and melt nose strips) were cut into small pieces to provide a representative sample for extraction. A fraction of 0.05 g of mask pieces or laden GFF was placed in a 15 mL glass tube. The sample was then extracted with 5 mL of hexane, followed by fortifying 100 ng each of deuterated internal standards. After ultrasonication at 100 kHz for 30 min and centrifugation at 3000 rpm for 15 min, the solvent layer was transferred into another glass tube, and the extraction step was then repeated. Before instrument analysis, the combined extraction solvent was concentrated to 1 mL under a gentle nitrogen stream, centrifuged at 10,000 rpm for 3 min, and then spun into a gas chromatographic glass vial.

Determination of target PAEs was conducted on an Agilent 7890A gas chromatography equipped with a 5977B mass spectrometry (GC-MS; Agilent Technologies, MA, USA) using an electron ion source and selected ion monitoring mode based on Cheng et al.’s study [24]. A DB-5MS column (Agilent; 30 m × 0.25 mm × 0.25 μm) was used for chromatographic separation of target chemicals. Further instrument parameters and detailed information of mass spectrometry are listed in Tables S3 and S4. Chromatograms of standards and a real mask sample are shown in Figure S2.

### 2.5. Microplastic Detection

The microplastics intercepted onto the GFF were observed and counted under a stereomicroscope (Sunny Optical Technology Co., Ltd., Ningbo, Zhejiang, China). To determine the exact membrane collecting microplastics, four types of membranes, including GFF, nylon filter, polytetrafluoroethylene filter, and mixed cellulose filter, which were commonly used for air sampling, were detected and compared the background of microplastic quantity. Except GFFs, 5–31 microplastics were observed on the latter three membranes (Table S5). At the same time, accounting for the non-plastic nature of GFF, which means low PAEs background concentration, GFF was finally chosen as the membrane for collecting microplastics and phthalates.

### 2.6. PAEs Exposure Assessment

Based on the measured concentrations in GFFs and facemasks, human exposure to PAEs via inhalation and dermal contact due to mask-wearing were calculated using the following equations [25]:

(1) $${\mathrm{EDI}}_{\mathrm{inh}}=\frac{m\times \frac{T_{\mathrm{inh}}}{T_{\mathrm{bre}}}}{\mathrm{BW}\times T}$$

where EDI_inh_ is estimated inhalation daily intake (ng/kg bw/day); m is PAE amount in GFF after a 6 h process (ng) (method A: 4 h + 2 h mass on GFF; method B: 6 h mass on GFF); $\frac{T_{\mathrm{inh}}}{T_{\mathrm{bre}}}$ is the proportion of the time taken to inhale during a full breath, the value is 1/3 (dimensionless) [26]; BW is the body weight of a person, 17.1 kg for a child, 60.6 kg for an adult [27,28]; and T is the exposure duration (1 day).

(2) $${\mathrm{EDI}}_{\mathrm{der}}=\frac{\frac{M}{V}\times k_{p-l}\times K_{\mathrm{ssl}-g}\times \mathrm{SA}\times T}{K_{\mathrm{cl}-g}\times \mathrm{BW}}$$

where EDI_der_ is estimated dermal daily intake (ng/kg bw/day); M is PAE amount measured in the mask (ng); V is mask volume (estimated to be 52.0 cm^3^) [29,30]; $k_{p-l}$ is the rate of chemicals transferring from skin surface lipid to blood (cm/h) (shown in Table S6, [25]; and $K_{\mathrm{ssl}-g}$ and $K_{\mathrm{cl}-g}$ are the partition coefficients (dimensionless) of PAEs between skin surface lipid and air, and between mask and air, respectively (Table S6, [31]). $K_{\mathrm{cl}-g}$ was omitted while calculating the ${\mathrm{EDI}}_{\mathrm{der}}$ of the air sample; SA is the contact surface area between skin and mask (estimated to be 166 cm^2^) [29]; and T is mask wearing time (assumed to be 6 h/day).

### 2.7. Quality Assurance and Quality Control

Prior to use, glass tubes and GFFs were baked at 450 °C for 4 h, and 1.5 mL plastic centrifugal tubes were cleaned twice with methanol to remove the background PAEs. A blank sample was analyzed with every 10 samples. Among the target chemicals, DNBP, DEHP, and DNOP were detected in procedural blanks ranging from 1.94 to 7.63 ng/mL, and these values were subtracted from reported concentrations in the present study. Two mask samples were prepared with each batch to evaluate the repeatability of the analytical method. The coefficient of variation of target chemicals in different batches of pooled mask samples ranged from 6.7–15.1%. The recoveries of the seven PAEs spiked into facemasks and GFFs were determined at two levels, i.e., 50 and 500 ng/g, and were in the range of 93–126% and 112–128% for masks, 51–108% and 86–128% for GFFs, respectively (Table S7). The limit of detection (LOD) and limit of quantification (LOQ) were defined as the concentration in matrix samples that generated signal-to-noise ratios of 3 and 10, respectively. The range of LODs was 0.002–3.41 ng/g for real samples (Table S7).

A cotton lab coat and nitrile gloves were worn to avoid microplastic contamination during the experiment. Furthermore, GFFs used for sampling were cleaned twice with Milli-Q water, and no microplastic could be detected after this step. After sampling, the suction cup was cleaned with Milli-Q water to ensure that all microplastics were transferred onto the membrane, and GFFs were stored in clean membrane wares. The masks and suction cups were covered with aluminum foil when the equipment was not switched off. During microplastic detection, the microscope was covered by a nylon bag.

### 2.8. Statistical Analysis

Qualitative software was used to integrate the peak area of the gas chromatogram of target chemicals. Values that were below LOD and between the LOD and LOQ were defined as zero and LOQ/√2, respectively, for statistical analysis. Statistical analyses, including Spearman’s correlation analysis, which gave correlation among 7 phthalates concentration in masks, ANOVA, which gave ∑_7_PAEs concentration difference among 5 types of masks, and a paired t-test analyzing the significant difference of PAEs collected in indoor and outdoor conditions, were performed using IBM SPSS Statistics 26.0. A *p*-value < 0.05 was statistically significant.

## 3. Results and Discussion

### 3.1. PAEs Residue Levels in Face Masks

Among the seven targeted PAEs in the present study, DNBP and DEHP were the predominant plasticizers and detected in all mask samples, with mean concentrations of 264 and 6804 ng/g, respectively (Table 1). This is in agreement with their large production and extensive use in melt-blown fabric and non-woven fabric of masks [9]. In contrast, the detected frequencies of DIBP, DEP, DMP, and BBZP ranged from 18–45%, and their mean concentrations were 805, 502, 558, and 51.2 ng/g, respectively. DNOP was not detected in any mask samples, which was consistent with the findings reported by Wang et al. [20]. The overall concentrations of the 7 PAEs (hereafter referred to as ∑_7_PAEs) in face masks ranged from 296 to 72,049 ng/g, with a median of 1401 ng/g. This result was consistent with those collected from China (median: 2050 ng/g), Europe (2890 ng/g), Japan (1459 ng/g), Korea (787 ng/g), and USA (1950 ng/g) [9,19]. In comparison to other skin-contact substances, ∑PAEs’ concentrations in facemasks were in the same order of magnitude with that of panty liners (∑_9_PAEs: 168–34,500 ng/g, median: 1830 ng/g) and pads (∑_9_PAEs: 205–11,200 ng/g, median: 362 ng/g) for females [32], infant clothes (∑_6_PAEs: 2290–51,900 ng/g, median: 4150 ng/g) [33], and children’s clothing (∑_6_PAEs: 1969–183,248 ng/g, median: 5579 ng/g) [25]. These results indicate that facemasks also represent an important source of PAE exposure in daily life and warrant further study.

With regard to the facemask type, we found that the fashion mask had the highest ∑_7_PAEs concentrations (median: 37,386 ng/g), which was two orders of magnitude higher than that in the surgical mask (367 ng/g) and N95 mask (577 ng/g) (Table S8). The other two types of facemasks, i.e., activated-carbon and cotton mask, contained similar residual levels of ∑_7_PAEs, with median values ranging from 5000–5689 ng/g. The various ∑_7_PAEs levels of masks may be due to different production standards and processes. Nevertheless, there was no significant difference among five types of masks (*p* > 0.05). Similar to our study, Arribas et al. [34] detected organophosphate esters (OPEs), another important class of plasticizers, in masks and found no statistical difference in OPE levels in common facemasks. This result may be related to similarities in raw materials used and limited facemask sample size in the present study [9].

Considering individual PAEs, DEHP and DNBP yielded the most significant contributions, accounting for 47.3% and 16.5%, respectively (Figure S3). It should be noted that DIBP yielded the largest contribution in cotton masks (79.8%), which may be related to the special materials. Although not statistically significant, there was a positive correlation between DNBP and DEHP (r = 0.52, *p* > 0.05). This may be attributed to the multiple purposes for these two primary PAEs. As a representative low molecular weight PAE, DNBP is often used as a solvent to maintain the color and smell of materials, while DEHP is indicated to soften polyvinyl chloride materials [35]. Furthermore, Vimalkumar et al. [19] found that DEHP clustered with non-phthalate plasticizers such as dibutyl sebacate and bis (2-ethylhexyl) adipate in facemasks by principal component analysis.

Phthalates are readily released from facemasks irresponsibly disposed of and believed to be a significant source of DEHP and other phthalates in the environment. An estimated 129 million masks (each weighing 4 g) were used every month worldwide during the COVID-19 pandemic in 2020 [5,29], suggesting that 5.2 × 10^5^ tons of masks were consumed every month. Based on the median concentration of ∑_7_PAEs measured in this study and assuming that 44% of the masks ended up as waste in the environment [36], 3415 tons of PAEs are calculated to have entered the environment. This is roughly equivalent to 0.04% of the annual production of PAEs, and the disposal of these masks is threatening to be a huge problem for the environment.

### 3.2. Microplastic Quantity in Inhalation Measurements

**Figure 2:**
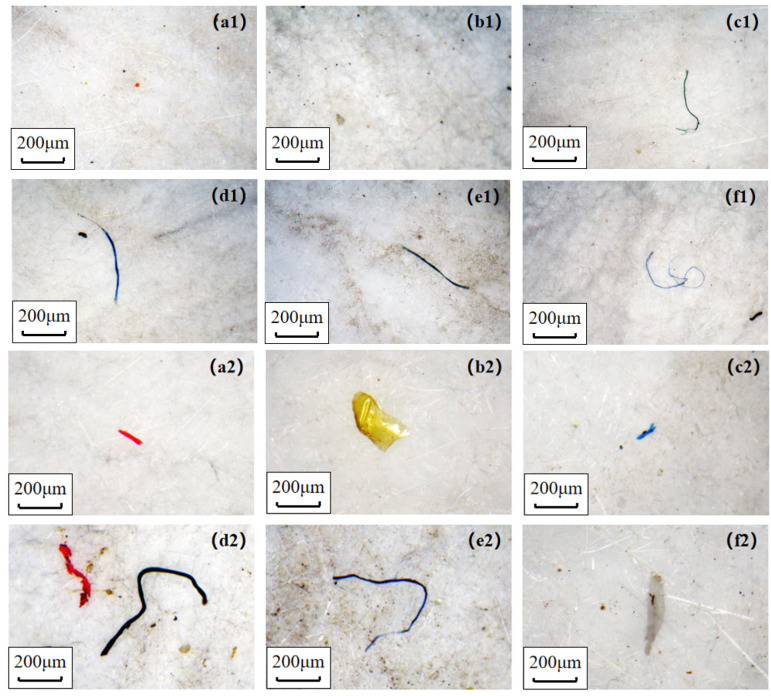
Microplastics detected on GFFs under indoor (**top**, (**a1**–**f1**)) and outdoor (**bottom**, (**a2**–**f2**)) conditions. (**a**–**f**): activated-carbon mask (AC), N95 mask (N95), surgical mask (SU), cotton mask (CO), fashion mask (FA), without mask (AIR), respectively.

As shown in Figure 2, microplastics retained by GFFs under indoor or outdoor conditions were divided into fragments and fibers, and they were counted separately over time. After 24 h simulating the release experiment, the amount of microplastics (sum of fibers and fragments) was 62–487 items/GFF. However, Li et al. counted 4000–30,000 microplastics after 24 h process, which is two magnitudes higher than our finding [18]. We thought that the disparity might be related to the different experiment environment (an indoor condition with lots of textiles in Li et al.) and count rules of microplastics. The microplastics number ranked in the following order: blank > cotton > activated-carbon > fashion > N95 > surgical and cotton > blank > activated-carbon > fashion > surgical > N95 under indoor and outdoor environments, respectively (Tables S9–S11). The largest number of microplastics in the blank sample and less microplastic in the N95 sample under indoor conditions was the same as the discovery of Li et al. [18]. This finding indicated that most masks could reduce respiratory exposure to microplastics compared to not wearing them, except for cotton masks. In this respect, the surgical masks and N95 masks yielded the best performance in reducing the inhalation risk of microplastics, for use either indoors or outdoors. Furthermore, microplastics were significantly higher outdoors (62–254 items/GFF) than those of indoors (139–487 items/GFF) after the whole release process, which was different from previous studies [37] reporting microplastics’ pollution in air was severer in indoor conditions. The discrepancy could be explained by different indoor conditions: apartments contain many textiles contributing a mass of fibers [37], whereas a laboratory environment does not provide a large source of textile fibers. It should be noted that we failed to analyze the chemical composition of microplastics and compared the blank sample and mask samples because of the limit of instruments, so we did not discuss the source (masks themselves or air) of microplastics or compare the release ability of different masks.

With regard to microplastic shape, fragmented items (68–86%) were significantly more than fibers (14–32%) in most mask samples except for cotton masks (49% vs. 50%) (Figure S4). The abundance of fibers at each point in time was the highest in cotton mask samples indoors and outdoors, reaching 119 and 245 items/GFF after 24 h release, which was 3.31 and 1.74 times higher in indoor and outdoor air samples, respectively (Table S9). Similarly, a study found that cotton masks released significantly more fibers (823) than surgical masks (85) when washing various types of masks in a washing machine [38]. This finding may be related to the loose structure of the cotton mask leading to a large amount of fiber released from the inner layer of this mask during inhalation [38,39]. In contrast, the abundance of fragmented microplastic in cotton masks was slightly lower than in indoor and outdoor air samples (Table S10), suggesting that cotton masks had a weak ability to protect against fragmented microplastics. Interestingly, the fiber amount detected in the activated carbon mask sample was more than the blank sample in the indoor environment, but the opposite findings were observed outdoors. This might be related to the larger amount of fiber in the air (Figure S4), as well as the stronger prevention and weaker release abilities of the activated carbon mask.

### 3.3. Microplastic Release Characteristics

**Figure 3:**
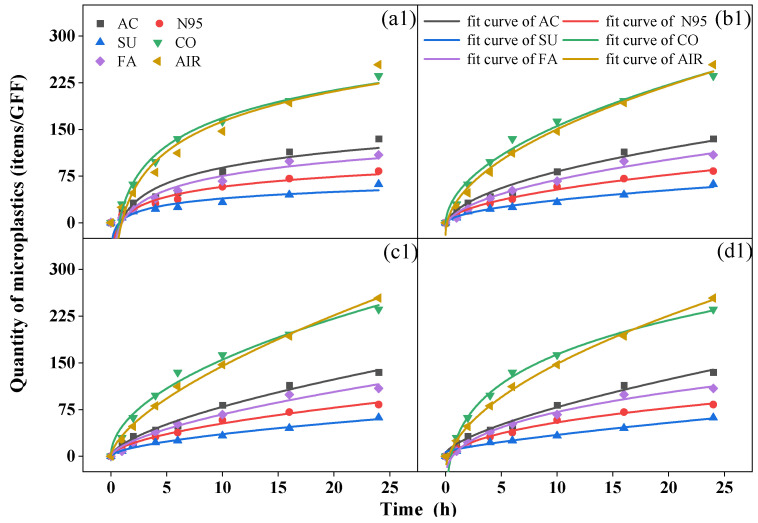

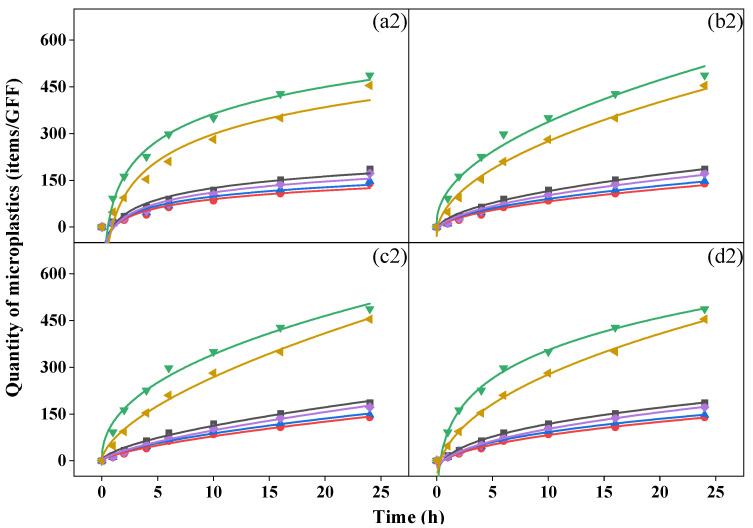
Fit curves of microplastics over time from (**a**) Elovich equation, (**b**) Parabolic diffusion equation, (**c**) Power function equation, and (**d**) modified Freundlich equation of activated-carbon mask sample (AC), N95 mask sample (N95), surgical mask sample (SU), cotton mask sample (CO), fashion mask sample (FA), and blank (AIR) in indoor (**top**, (**a1**–**d1**)) and outdoor (**bottom**, (**a2**–**d2**)) conditions.

During the 24 h period, a biphasic release pattern was observed with rapid release in the first six hours, followed by slower release indoors and outdoors. Although it could not be precisely determined whether the microplastics collected came from the mask or the atmosphere, the variations in the quantity over time were similar to the release trend of the microplastics in the water environment of the mask. Similar equations (Equations (3)–(6)) can be used to fit the dynamic characteristics [40,41] (Figure 3).

Elovich equation:

(3) $$Q_{t}=a+\mathrm{blnt}$$

Parabolic diffusion equation:

(4) $$Q_{t}=a\;+{\;\mathrm{bt}}^{0.5}\;$$

Power function equation:

(5) $$Q_{t}={\mathrm{bt}}^{c}\;$$

Modified-Freundlich:

(6) $$Q_{t}=a\;+{\;\mathrm{bt}}^{c}\;$$

where t is the release time (h); Q_t_ is the quantity of microplastics at the time t (item/GFF); a is the initial quantity of microplastics (item/GFF); and b and c is the rate constant.

We analyzed relevant parameters for the four fitting equations (Table S12 for indoor conditions and Table S13 for outdoor conditions) and found that all R^2^ values were > 0.92, indicating the proper equations were chosen, and the modified Freundlich equation yielded the best fitting results (R^2^ > 0.98). A c value less than 1 in the power function equation and modified Freundlich equation suggested that the quantity of microplastics collected by GFFs decreased exponentially per unit time [42]. The b value in the four equations reflects the rate of increase in microplastics; a greater b was associated with a greater increase in rate [40]. In all samples, the cotton mask yielded the largest b value in both conditions, but the surgical mask and N95 mask showed the smallest b indoors and outdoors, respectively, similar to the quantity of microplastics we discussed before. This finding proved that the number of microplastics collected during release was closely related to the type of mask. This is inconsistent with the conclusion reached by Liang et al. [40], who reported no relationship between the release of microplastics in water and the type of mask. Moreover, Wu et al. [43] reported that the release of microplastics from surgical masks was greater than from ordinary and filtering facepiece masks. Indeed, during the process of this experiment, the mask not only released microplastics but also blocked microplastics from the air to various degrees.

We analyzed the kinetic characteristics of microplastics, classified into five groups, 20–30, 30–100, 100–500, 500–1000, and >1000 μm, according to their size (Figure S5). As shown in Figure S6, in indoor and outdoor environments, microplastics with small sizes, such as 20–30 μm (indoor: 16–63%; outdoor: 3–29%) and 30–100 μm (indoor: 21–46%; outdoor: 24–60%), were the dominant groups during the inhalation experiment except for the cotton mask sample, while the 100–500 μm group was mostly collected by cotton mask. Interestingly, microplastics > 500 μm almost stopped increasing after 10 h since large-sized microplastics (mostly fibers) on the mask surface were released more easily and quickly at the beginning of the process [40], but in the subsequent 18 h, the large-sized microplastics in the air could be blocked by the mask, and fibers inside the mask are difficult to release due to their interweaving.

### 3.4. PAEs on GFFs and Associations with Microplastics

**Figure 4:**
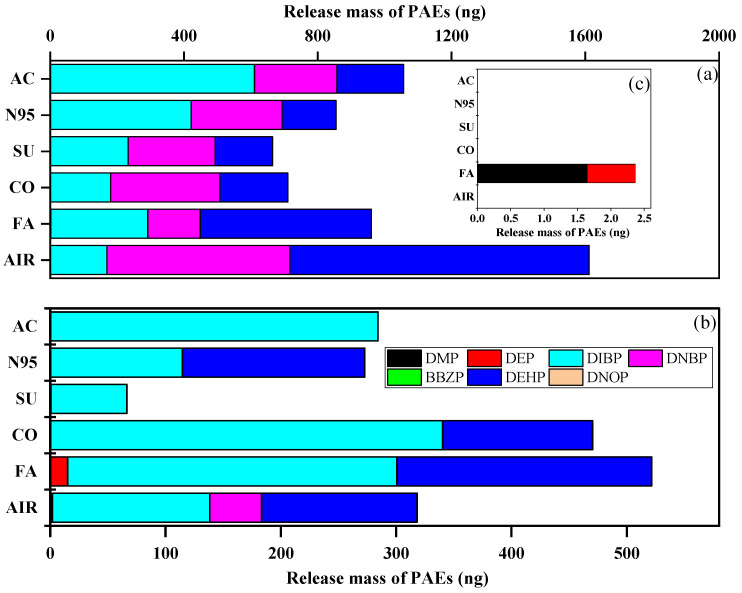
Average 24 h collected mass of each targeted compound of activated-carbon mask sample (AC), N95 mask sample (N95), surgical mask sample (SU), cotton mask sample (CO), fashion mask sample (FA), and blank (AIR) under (**a**) indoors and (**b**) outdoors. (**c**) is the enlarged drawing of DMP and DEP in indoor condition.

We detected released PAEs on GFFs during the inhalation experiment and compared the indoor and outdoor mass of the ∑_7_PAEs (Figure S7). Interestingly, the mass of ∑_7_PAEs (mean: 999 ng vs. 411 ng, *p* < 0.05) was significantly higher indoors than outdoors. This result was consistent with the literature [44,45], indicating that indoor environments showed higher exposure sources than outdoors. After the whole inhalation experiment, the ∑_7_PAE mass of all GFF samples were as follows: blank > activated-carbon > fashion > N95 > cotton > surgical mask and fashion > cotton > blank > N95 > activated-carbon > surgical mask indoors and outdoors, respectively (Figure 4). This data indicated wearing masks could decrease human exposure to PAEs (i.e., acting as a “protector”) indoors, but in outdoor conditions, their effects differed and were affected by types.

Interestingly, after further analysis of each compound’s average mass (as shown in Figure 4), we found that mask-wearing could increase the inhalation dose of DIBP (except for surgical masks outdoors) but reduce that of DNBP and DEHP, which can be attributed to their logK_OA_. DIBP had the lowest value and could easily enter the air and then be trapped by GFFs during “inhalation” [31]. Moreover, the mass of DEP in the fashion mask sample was higher than in indoor and outdoor air samples, which may be caused by the large amount of DEP (1421 ng/g) released from fashion masks during the inhalation experiment. Overall, masks can reduce the inhalation dose of ∑_7_PAEs but can release DIBP, while fashion masks represent an important source of DEP.

During the “inhalation” process, the mass of ∑_7_PAEs increased gradually; the same equation as microplastics was not used considering the better linearity (R^2^ > 0.93) of PAEs except surgical and cotton mask samples outdoors, which was related to more complex flux outdoors. However, there was still a significant correlation between microplastics and PAEs (indoor: r = 0.852, *p* < 0.01; outdoor: r = 0.620, *p* < 0.01, as shown in Figure S8), and smaller microplastic size corresponded to a more severe PAE load (Table S14), as microplastics with smaller size could carry PAEs from processed materials because of the larger specific surface area [46].

### 3.5. Exposure Estimation

**Figure 5:**
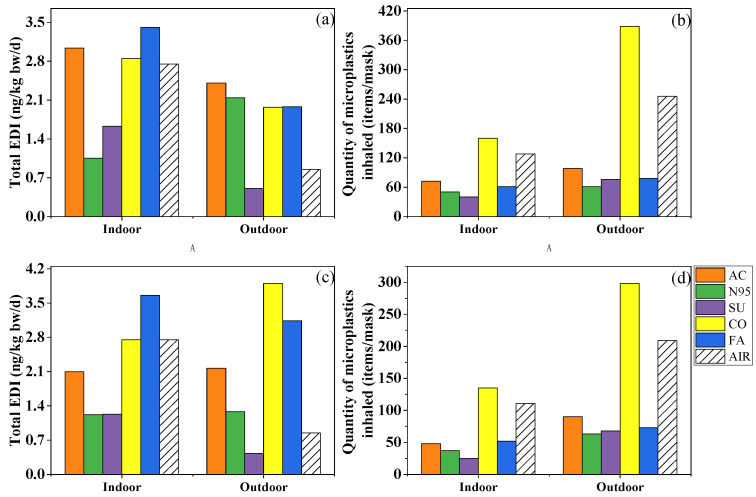
Total estimated daily intake of phthalates (**a**,**c**) and quantity of microplastics inhaled (**b**,**d**) by an adult while wearing activated-carbon mask (AC), N95 mask (N95), surgical mask (SU), cotton mask (CO), fashion mask (FA), and wearing no mask (AIR) in indoor and outdoor conditions. (**a**,**b**) simulated the condition in which the mask was changed after 4 h and kept for later 2 h, while (**c**,**d**) were based on the mass of phthalates and number of microplastics collected after 6 h.

In recent years, several studies which analyzed the PAE exposure risk when wearing masks yielded comparable results despite using different methodologies. In the present study, the EDI_inh_ of the ∑_7_PAEs ranged from 3.60 to 8.62 and from 1.47 to 7.49 ng/kg bw/d indoors and outdoors for children, and from 1.02 to 2.43 and from 0.493 to 2.11 ng/kg bw/d for adults, which was comparable with the EDI values (5.14 and 2.02 ng/kg bw/d for children and adults, respectively) calculated by Vimalkumar et al. [19]. Wearing fashion, cotton and activated-carbon masks indoors and outdoors increased the inhalation risk of PAEs, while surgical masks could reduce that of PAEs (Figure 5a,b). Furthermore, Wang et al. [20] calculated the EDI_inh_s caused by wearing surgical and N95 masks and found that the EDI of the former was almost ten-fold less than the latter, indicating the safer nature of the surgical mask.

Taking dermal exposure into consideration, given PAEs’ high concentration in fashion masks (2724–72,049 ng/g), the EDI_der_ of this mask reached 0.98 and 3.5 ng/kg bw/d for adults and children, respectively, while the level of N95 and surgical masks was smaller than 0.1 ng/kg bw/d (Figure S9b). However, a study reported that the dermal risk of 11 PAEs caused by wearing masks ranged from 3.71 to 639 ng/kg bw/d [9], which was significantly higher than in our study, while their PAE concentration (115–37,700 ng/g) in masks was comparable to our results (296–72,049 ng/g), which may be related to the different exposure scenarios and parameters selected for skin penetration. Xie et al. [9] multiplied the mass of PAEs in masks and the human body absorption rate (20%) to calculate the dermal risk. Nonetheless, in our study, after considering the equilibrium of PAEs among mask, air, and skin surface and the rate of chemicals’ transfer from skin surface lipid to blood, the equivalent “absorption rate” was only 5.30 × 10^−5^–6.34 × 10^−3^% for individual phthalates, four to six orders of magnitude lower than the previous study, resulting in lower EDI_der_.

After summing up the two exposure approaches (EDI_total_), more types of masks could potentially be considerable sources of PAEs. Among the five types, fashion masks brought the most serious risk, followed by cotton and activated-carbon masks, while the surgical mask still played a protective role (Figure 5a). The EDI_total_ was 1.05–12.1 ng/kg bw/d indoors and 0.507–7.03 ng/kg bw/d outdoors, which was significantly lower than the EDI of dietary exposure (1.03–4.68 µg/kg bw/d) and reference dose (R*f*D) of a single PAE (R*f*D = 20–1000 µg/kg bw/d) [47,48,49,50,51,52,53], indicating human exposure to PAEs via masks may not pose a potential health risk. However, it should be borne in mind that fashion masks represent an important source of PAEs indoors and outdoors.

Different from the complex and dependent risk assessments of PAEs, microplastics’ risk mainly appeared on one mask whether in indoor and outdoor conditions. Although nanoplastics smaller than 100 nm can easily enter the lungs due to their small size, fibers as long as 2475 μm have been documented in human lung tissues [54]. Based on the amount of 20–2475 μm microplastics trapped by the filter membrane (Figure 5b), after 6 h of wearing, 40–160 and 61–389 microplastics may be inhaled in indoor and outdoor environments, respectively, and cotton masks represent the most important source of microplastics. However, due to limitations in measurement instruments, this study failed to quantify nanoplastics. A previous study observed that an amount of approximately 10^9^ of nanoplastic could be released when the mask was oscillated in a water environment for 4 h [55]. Although it differs from the breathing environment, the massive release of nanoplastics is still worthy of attention and needs further research and discussion.

We further analyzed the difference between Method A and Method B while calculating the exposure dose of microplastics and PAEs (Figure 5a–d). Briefly speaking, the total exposure dose of PAEs of most masks and the inhaled microplastics of all masks increased. The EDI_total_ of fashion masks decreased from 3.66 to 3.41 ng/kg bw/d indoors and, in opposite fashion, from 3.14 to 1.98 ng/kg bw/d outdoors. As for different masks’ roles, activated-carbon, cotton, and fashion masks increased PAE exposure whether in indoor or outdoor conditions, but cotton masks were still the only mask increasing the inhalation amount of microplastics after changing the masks’ usage. In a word, different masks’ usage could result in different exposure doses; on one hand, it was suggested to change masks after 4 h to avoid viruses, but on the other hand, changing masks too often might increase some exposure to pollutants, microplastics at least.

## 4. Conclusions

In this study, we discussed the role of five widely used masks on microplastic and phthalate plasticizer exposure with respect to environmental health. We observed considerable pollution of phthalates in widely used masks, with the lowest concentration in surgical masks, and the highest levels in fashion masks. Among seven phthalates, DNBP and DEHP were detected in each mask sample. During the inhalation experiment, microplastics increased faster in the first 6 h, and fragments and small-size microplastics accounted for the largest. For our main focus, surgical masks play a protective role, while wearing activated carbon, cotton, and fashion masks in indoor and outdoor environments increases human exposure to PAEs. Additionally, wearing cotton masks resulted in a larger inhalation risk than wearing no mask, while other types of masks acted as protectors against microplastics.

However, we call for more studies and development based on some limitations. The limited number of masks was not enough to analyze the statistical significance of various masks ∑_7_PAE concentrations and to stand for exact exposure characteristics of various types of masks while wearing; secondly, our release experiment, which was conducted under laboratory conditions, only focused on the inhalation process, and exhalation was not concerned although inspiration–expiration ratio was considered to reduce deviation while calculating EDI_inh_; meanwhile, the simulation inhalation process, especially the air flow rate, was different from a real situation considering masks’ distinctive fluid resistance; finally, on account of the limit of instruments, we did not obtain the chemical composition of microplastics collected, and the fibers of the cotton mask sample were assumed to be microplastics, so there might be an overestimation of the microplastic exposure.

Even so, our study corroborates that cotton and fashion masks are important sources of human exposure to microplastics and phthalates, highlighting that it is not recommended to wear these two masks unnecessarily in daily life to reduce exposure. In contrast, the surgical mask is a great choice against microplastics and phthalates, as well as the COVID-19 virus.

## Figures and Tables

**Table 1:** Concentrations (ng/g) and detection frequencies (DFs, %) of phthalates in mask samples (*n* = 11) purchased from China market.

Compounds ^1^	DFs (%)	Mean	Median	Minimum	Maximum
DMP	18	558	<LOD	<LOD	6093
DEP	27	502	<LOD	<LOD	2189
DIBP	45	805	<LOD	<LOD	7982
**DNBP**	**100**	**264**	**294**	**85.5**	**418**
BBZP	18	51.2	<LOD	<LOD	419
**DEHP**	**100**	**6804**	**316**	**63.5**	**69,496**
**∑_7_PAEs**	**100**	**8986**	**1401**	**296**	**72,049**

^1^ DNOP was rarely detected in mask samples. Bold represented important compounds with DF = 100%.

## Data Availability

The data presented in this study are available upon request from the corresponding authors. The data are not publicity available due to the very large sizes of the chromatographic files.

## Supplementary Materials

The following supporting information can be downloaded at: https://www.mdpi.com/article/10.3390/toxics11020087/s1, Figure S1: 5 types of facemasks we used in the research. The first row from left to right are cotton mask, N95 mask, and fashion mask, respectively. The masks below are activated-carbon mask (left) and surgical mask (right). Figure S2: Chromatograms of 7 phthalates in this study; Figure S3: Composition of detected PAEs in activated-carbon mask (AC), N95 mask (N95), surgical mask (SU), cotton mask (CO), and fashion masks (FA); Figure S4: Quantity of fragments and fibrous microplastics of activated-carbon mask (AC), N95 mask (N95), surgical mask (SU), cotton mask (CO), fashion masks (FA) in indoor (IN) and outdoor (OUT) conditions after 24 h of suction; Figure S5: Size proportion of all microplastics after 24 h of suction in (a) indoor and (b) outdoor conditions; Figure S6: Five groups of microplastics collected from activated-carbon mask sample (AC), N95 mask sample (N95), surgical mask sample (SU), cotton mask sample (CO), fashion mask sample (FA), and blank sample (AIR) in indoor (top) and outdoor (bottom) conditions; Figure S7: Mass of phthalates in GFFs from indoor (IN) and outdoor (OUT) conditions after 24 h; Figure S8: Linear correlation of MPs quantity and ∑_7_PAEs mass in (a) indoor and (b) outdoor conditions; Figure S9: Inhalation and dermal estimated daily intake of phthalates of an adult (a, c) and a child (b, d) and a child’s total estimated daily intake (e) while wearing activated-carbon mask (AC), N95 mask (N95), surgical mask (SU), cotton mask (CO), fashion mask (FA), and wearing no mask (AIR) in indoor and outdoor conditions. Table S1: Chemical properties of target PAE compounds; Table S2: Layers and materials of five types of masks in this study; Table S3: GC-MS Instrument parameters; Table S4: Mass spectrometry ions selected, retention time, and correlation coefficient in the analysis of 7 phthalates in this study; Table S5: Microplastics detected on different blank filters. Table S6: Estimated parameter values of dermal exposure to PAEs; Table S7: Recoveries, limits of detection (LODs), and quantification (LOQs) of GC-MS analysis of PAEs in mask and GFF samples; Table S8: Background concentration of phthalates (PAEs) in different types of masks (ng/g); Table S9: Quantity of fiber-like microplastics on GFFs indoors and outdoors (items/GFF) during inhalation; Table S10: Quantity of fragmented microplastics on GFFs indoors and outdoors (items/GFF) during inhalation; Table S11: Quantity of microplastics on GFFs indoors and outdoors (items/GFF) during inhalation; Table S12: Parameters and R^2^ of each fitting model of microplastics in indoor conditions; Table S13: Parameters and R^2^ of each fitting model of microplastics in outdoor conditions; Table S14: Correlation parameters of microplastic size and ∑_7_PAE mass in indoor and outdoor conditions.
