# Supplementary material for: Face Mask: As a Source or Protector of Human Exposure to Microplastics and Phthalate Plasticizers?
Source: Toxics. 2023 Jan 17;11(2):87. doi: 10.3390/toxics11020087 (PMC9967050; doi:10.3390/toxics11020087)
Supplement: Supplementary file 1 [file toxics-11-00087-s001.zip › toxics-2075938-supplementary.pdf]

toxics

Supplementary Materials: Face Mask: As a Source or Protector of Human Exposure to Microplastics and Phthalate Plasticizers?

Table S1. Chemical properties of target phthalate compounds.

Compounds	Abbreviation	CAS	Formula	Molecular Weight (g/mol)	Structure	$LogK_{ow}$
Dimethyl phthalate	DMP	131-11-3	$C_{10}H_{10}O_4$	194.18	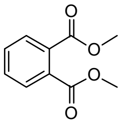	1.61
Diethyl phthalate	DEP	84-66-2	$C_{12}H_{14}O_4$	222.24	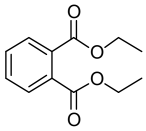	2.54
Di-iso-butyl phthalate	DIBP	84-69-5	$C_{16}H_{22}O_4$	278.34	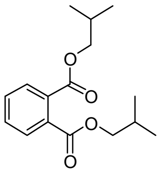	4.27
Dibutyl phthalate	DNBP	84-74-2	$C_{16}H_{22}O_4$	278.34	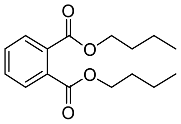	4.27
Butyl benzyl phthalate	BBZP	85-68-7	$C_{19}H_{20}O_4$	312.36	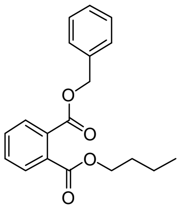	4.70
Di(2-ethylhexyl) phthalate	DEHP	117-81-7	$C_{24}H_{38}O_4$	390.56	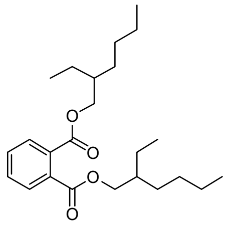	7.73
Di-n-octyl phthalate	DNOP	117-84-0	$C_{24}H_{38}O_4$	390.5561	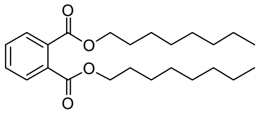	7.73

Table S2. Layers and materials of five types of masks in this study.

Serial	Layers and materials (from outside to inside)
	**Activated carbon mask**
M1	4 layers：Non-woven fabric, activated carbon filter, melt-blown cloth filter, nonwoven fabric
M2	4 layers：Waterproof non-woven fabric, activated carbon deodorant, melt-blown cloth filter, skin-friendly non-woven fabric
	**N95 mask**
M3	4 layers: Medical non-woven fabric, melt-blown fabric, melt-blown fabric, medical non-woven fabric
M4	4 layers: Non-woven fabric, melt-blown fabric, melt-blown fabric, skin-friendly non-woven fabric
M5	4 layers: Skin-friendly non-woven cloth, filter melt-blown cloth, high-quality melt-blown cloth; Skin-friendly non-woven fabric
	**Medical surgical mask**
M6	3 layers: Non-woven fabric, melt-blown fabric, activated carbon polypropylene non-woven fabric
M7	3 layers: Non-woven fabric, melt-blown cloth, non-woven fabric
	**Cotton mask**
M8	2 layers: 100% cotton, 98% polyester +2% spandex
M9	4 layers: Cotton, non-woven fabric, polymer layer, cotton
	**Fashion mask**
M10	1 layer: Polyurethane
M11	1 layer: Polyurethane

Table S3. GC-MS instrument parameters for phthalate measurements.

GC (7890A – Agilent technology)	
Carrier gas	Helium (99.999% pure) 1.2 mL/min
Oven temperature program	80 °C (1 min) → 200 °C (12 °C/min held for 1 min) → 300 °C (10 °C/min held for 2 min)
Injection volume	1 μL
MSD (5977B - Agilent technology)	
Injector temperature	250 °C
Ion source temperature	230 °C

Table S4. Mass spectrometry ions selected, retention time and correlation coefficient in the analysis of 7 phthalates in this study.

Compounds	Quantitative ion (*m*/*z*)	Quantification ion (*m*/*z*)	Retention time (min)	R^2
**DMP**	163	77	8.692	1.000
**DEP**	149	91/105/177	9.684	1.000
**DIBP**	149	104/188	11.818	1.000
**DNBP**	149	104	12.632	1.000
**BBZP**	149	206	16.117	1.000
**DEHP**	149	167	17.762	1.000
**DNOP**	149	167	20.169	0.999

Table S5. Microplastics detected on different blank filters.

Types of filters	Microplastics quantity
Glass fiber filter	4
nylon filter	7
polytetrafluoroethylene filter	5
mixed cellulose filter	31

Table S6. Estimated parameter values of dermal exposure about PAEs.

	DMP	DEP	DIBP	DNBP	BBZP	DEHP
$k_{p\text{-}l}$（cm/h）	1.82E-06	1.82E-06	1.82E-06	1.78E-06	9.60E-08	9.60E-08
$K_{ssl\text{-}g}$	2.20E+07	2.20E+07	2.20E+08	2.20E+08	2.20E+08	2.20E+08
$K_{cl\text{-}g}$	6.31E+05	6.31E+05	6.31E+06	1.58E+07	3.98E+07	2.51E+07

Table S7. Recoveries, limits of detection (LODs) and quantification (LOQs) of GC-MS analysis of PAEs in mask and GFF samples.

Analytes	Masks				GFFs			
	Recovery-50ng/g (%)	Recovery-500ng/g (%)	LOD ng/g	LOQ ng/g	Recovery-50ng/g (%)	Recovery-500ng/g (%)	LOD ng/g	LOQ ng/g
DMP	106	121	0.808	2.79	51	86	0.18	1.31
DEP	116	128	0.742	2.14	78	104	0.034	0.113
DIBP	109	125	0.073	0.245	106	128	0.002	0.006
DNBP	126	120	0.521	1.73	108	124	0.50	1.66
BBZP	93	112	2.60	8.40	98	122	2.6	8.40
DEHP	104	120	2.55	8.16	103	126	2.55	8.16
DNOP	101	113	3.41	10.8	102	114	3.41	10.8

Table S8. Background concentration of phthalates (PAEs) in different types of masks (ng/g).

	DMP	DEP	DIBP	DNBP	BBZP	DEHP	DNOP	$\sum_7$PAEs
	**Activated-carbon mask**							
M1	＜LOD	＜LOD	41.5	418	＜LOD	941	＜LOD	1401
M2	6093	1918	＜LOD	333	＜LOD	1632	＜LOD	9977
	**N95 mask**							
M3	＜LOD	＜LOD	474	385	＜LOD	223	＜LOD	1083
M4	＜LOD	＜LOD	＜LOD	85.5	＜LOD	211	＜LOD	296
M5	＜LOD	＜LOD	＜LOD	261	＜LOD	316	＜LOD	577
	**Surgical mask**							
M6	＜LOD	＜LOD	＜LOD	103	＜LOD	291	＜LOD	394
M7	＜LOD	＜LOD	＜LOD	131	＜LOD	209	＜LOD	341
	**Cotton mask**							
M8	＜LOD	＜LOD	7982	254	＜LOD	354	＜LOD	8590
M9	＜LOD	＜LOD	＜LOD	304	＜LOD	1106	＜LOD	1411
	**Fashion mask**							
M10	＜LOD	2189	33.8	294	143	63.5	＜LOD	2724
M11	48.8	1421	325	339	419	69496	＜LOD	72049

Table S9. Quantity of fiber-like microplastics on GFFs indoors and outdoors (items/GFF) during inhalation.

	Indoor						Outdoor					
	AC	N95	SU	CO	FA	AIR	AC	N95	SU	CO	FA	AIR
1 h	8	3	2	17	1	6	6	3	2	59	4	16
2 h	11	7	6	40	4	8	8	6	7	103	8	29
4 h	15	11	6	56	10	13	9	11	11	121	15	47
**6 h**	**21**	**13**	**8**	**74**	**13**	**19**	**16**	**16**	**21**	**156**	**16**	**64**
10 h	27	14	9	86	16	24	20	20	28	190	20	85
16 h	32	19	13	101	22	29	27	27	39	223	26	103
24 h	41	21	15	119	23	36	32	43	47	245	30	141

Table S10. Quantity of fragmented microplastics on GFFs indoors and outdoors (items/GFF) during inhalation.

	Indoor						Outdoor					
	**AC**	**N95**	**SU**	**CO**	**FA**	**AIR**	**AC**	**N95**	**SU**	**CO**	**FA**	**AIR**
1 h	13	9	6	13	7	19	11	7	9	33	8	32
2 h	21	12	12	22	18	40	26	16	23	60	19	65
4 h	27	20	16	42	29	68	55	28	35	105	36	106
**6 h**	**28**	**25**	**17**	**61**	**39**	**93**	**74**	**47**	**47**	**142**	**57**	**146**
10 h	55	44	24	77	51	123	99	64	67	160	84	196
16 h	82	52	32	95	77	164	125	80	80	205	111	247
24 h	94	62	47	117	86	218	154	96	100	242	142	313

Table S11. Quantity of microplastics on GFFs indoors and outdoors (items/GFF) during inhalation.

	Indoor						Outdoor					
	**AC**	**N95**	**SU**	**CO**	**FA**	**AIR**	**AC**	**N95**	**SU**	**CO**	**FA**	**AIR**
1 h	21	12	8	30	8	25	17	10	11	92	12	48
2 h	32	19	18	62	22	48	34	22	30	163	27	94
4 h	42	31	22	98	39	81	64	39	46	226	51	153
6 h	**49**	**38**	**25**	**135**	**52**	**112**	**90**	**63**	**68**	**298**	**73**	**210**
10 h	82	58	33	163	67	147	119	84	95	350	104	281
16 h	114	71	45	196	99	193	152	107	119	428	137	350
24 h	135	83	62	236	109	254	186	139	147	487	172	454

Table S12. Parameters and R^2 of each fitting model of microplastics in indoor condition.

	**Elovich**			**Parabolic**			**Power-function**			**Modified-Freundlich**			
	a	b	*R2*	a	b	*R2*	b	c	*R2*	a	b	c	*R2*
AC	5.02	36.3	0.921	-8.52	28.7	0.977	18.4	0.634	0.989	4.34	15.6	0.679	0.986
N95	4.54	23.1	0.970	-3.81	18.1	0.989	14.3	0.566	0.990	-18.6	29.1	0.398	0.993
SU	4.20	15.1	0.925	-2.30	12.2	0.979	9.10	0.592	0.986	6.52	4.75	0.767	0.985
CO	19.9	64.4	0.992	-4.85	50.7	0.986	47.7	0.512	0.985	-187	216	0.21	0.997
FA	-0.0480	32.7	0.972	-8.84	24.6	0.977	16.1	0.620	0.981	-42.9	50.5	0.353	0.990
AIR	1.98	69.8	0.958	-19.1	53.5	0.987	32.9	0.644	0.997	-25.4	51.1	0.531	0.998

Table S13. Parameters and R^2 of each fitting model of microplastics in outdoor condition.

	**Elovich**			**Parabolic**			**Power function**			**Modified-Freundlich**			
	a	b	*R2*	a	b	*R2*	b	c	*R2*	a	b	c	*R2*
AC	1.58	53.7	0.977	-13.7	40.8	0.986	27.4	0.613	0.990	-63.2	77.9	0.367	0.999
N95	-3.51	40.3	0.962	-13.4	30.2	0.978	16.6	0.674	0.990	-30.2	38.7	0.463	0.996
SU	-0.168	42.6	0.974	-11.8	32.2	0.984	20.6	0.628	0.989	-45.0	55.8	0.390	0.997
CO	76.0	125	0.994	12.4	103	0.985	122	0.445	0.990	-402	494	0.185	0.997
FA	-5.08	50.4	0.963	-17.3	37.7	0.979	20.3	0.681	0.993	-36.2	46.5	0.473	0.999
AIR	11.3	125	0.968	-28.7	96.1	0.990	66.0	0.609	0.995	-75.0	123	0.455	0.998

Table S14. Correlation parameters of microplastic size and $\sum_7$PAE mass in indoor and outdoor conditions.

**Size group**	**20 – 30 μm**	**30 – 100 μm**	**100 – 500 μm**	**500 – 1000 μm**	**>1000 μm**
indoor	**0.901****	0.904**	0.639**	0.650 **	0.500 **
outdoor	**0.818****	0.705**	0.455**	0.433**	0.264 ns

** $p < 0.01$; ns: no significancy.

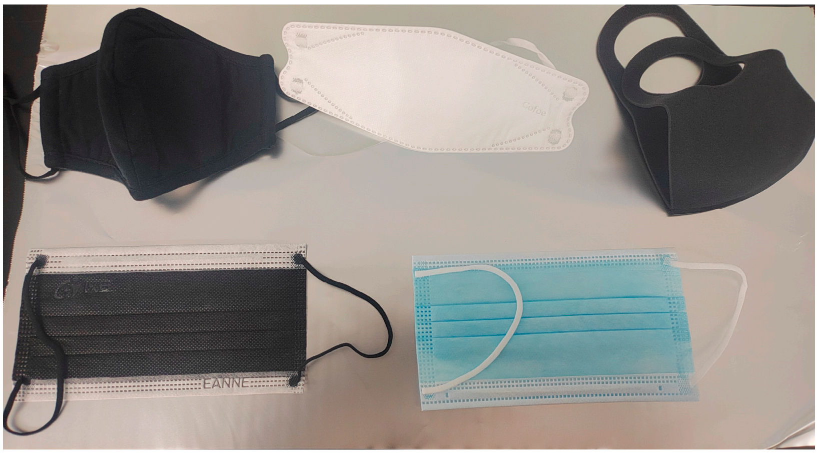

Figure S1. Five types of facemasks we used in the research. The first row from left to right are cotton mask, N95 mask, fashion mask, respectively. The masks below are activated-carbon mask (left) and surgical mask(right).

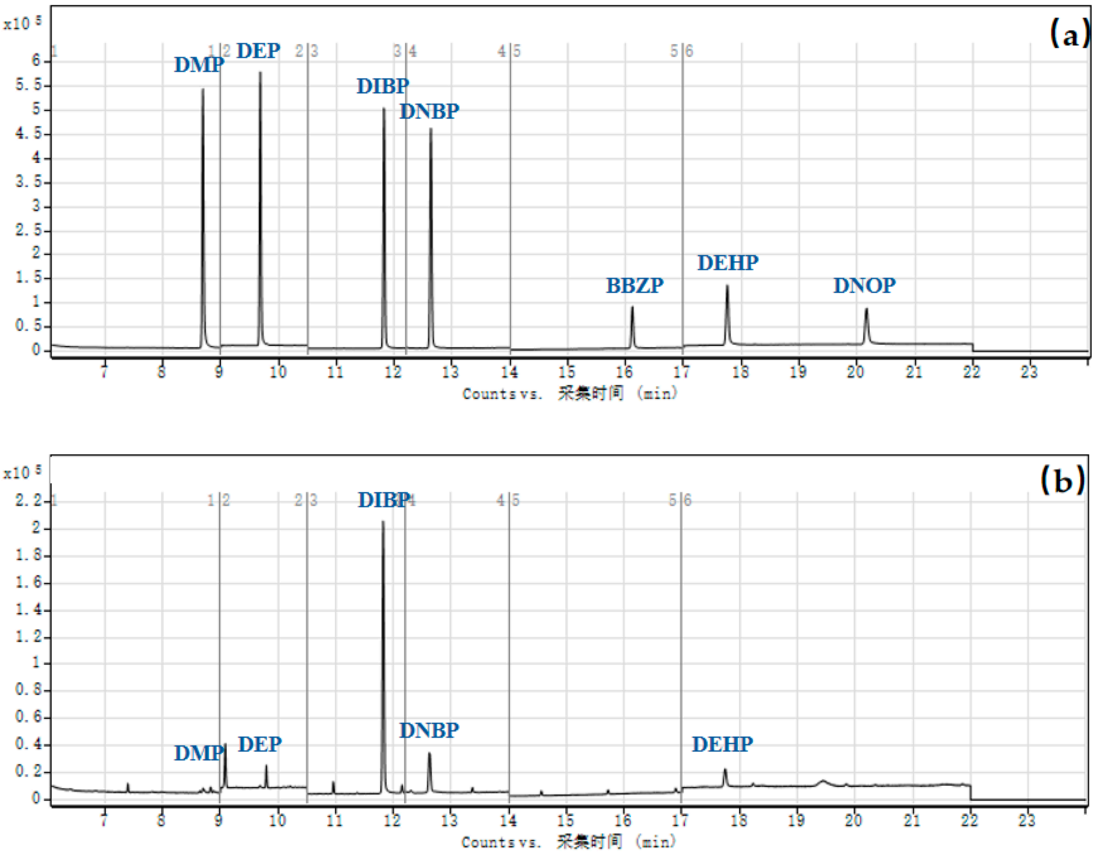

Figure S2. Chromatograms of 7 phthalates in this study. (a): Standard sample; (b): practical sample.

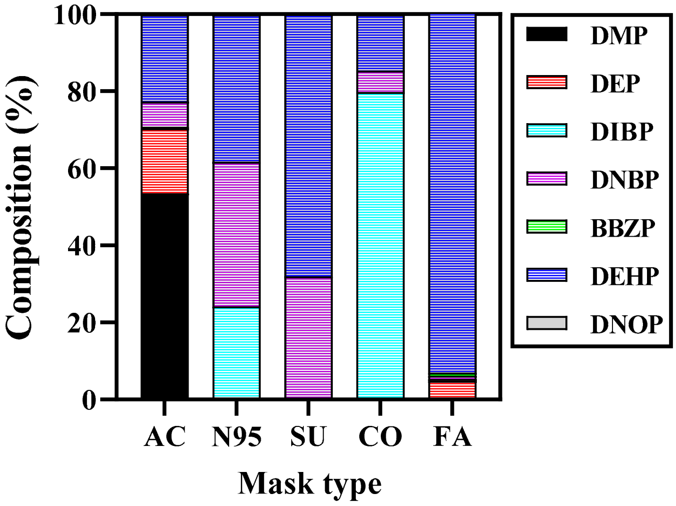

Figure S3. Composition of detected PAEs in activated-carbon mask (AC), N95 mask (N95), surgical mask (SU), cotton mask (CO), fashion masks (FA).

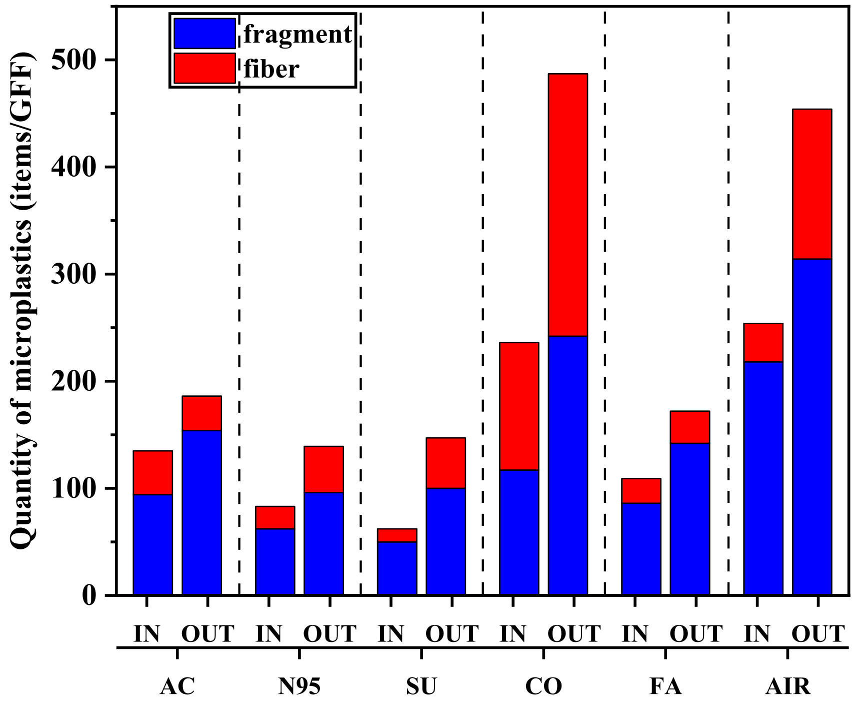

Figure S4. Quantity of fragments and fibrous microplastics of activated-carbon mask (AC), N95 mask (N95), surgical mask (SU), cotton mask (CO), fashion masks (FA) in indoor (IN) and outdoor (OUT) conditions after 24 hours' suction.

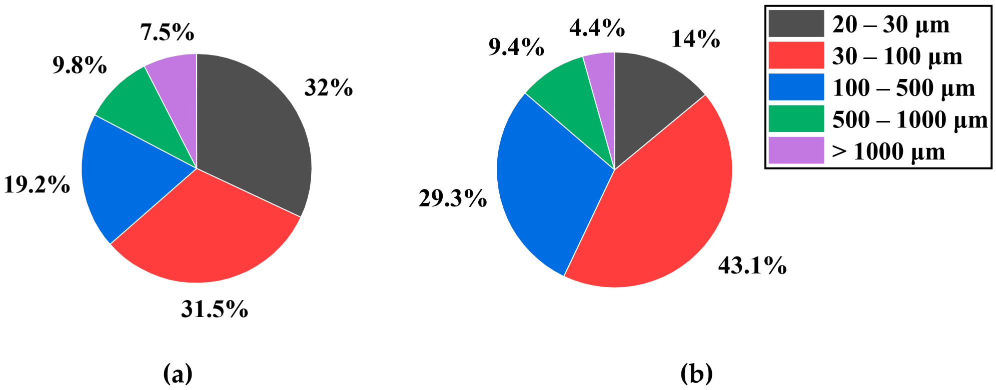

Figure S5. Size proportion of all microplastics after 24 hours' suction in (a) indoor and (b) outdoor conditions.

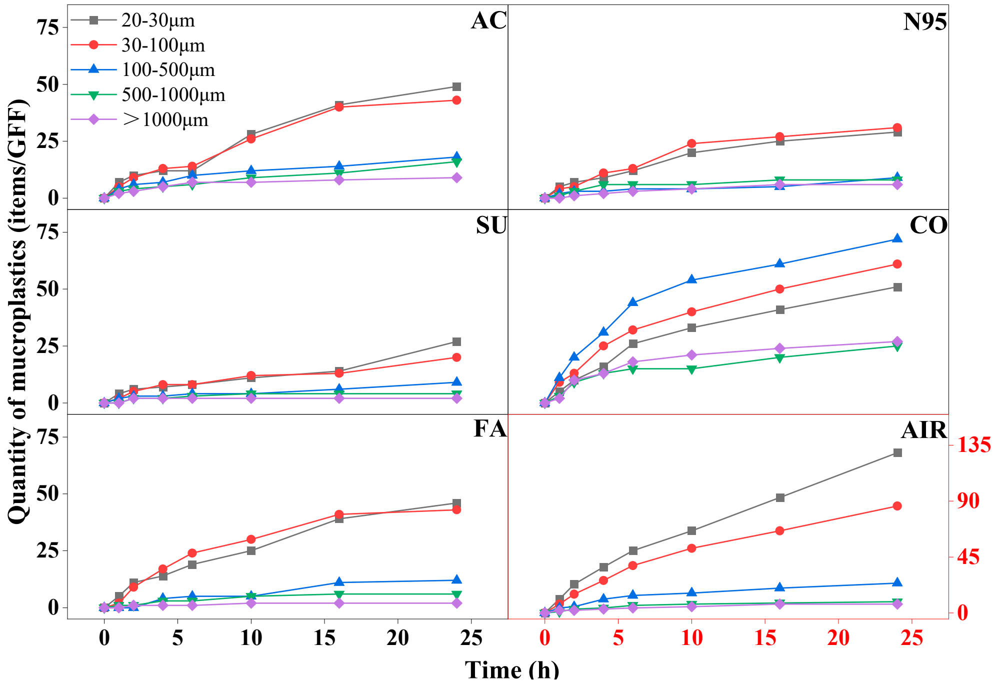

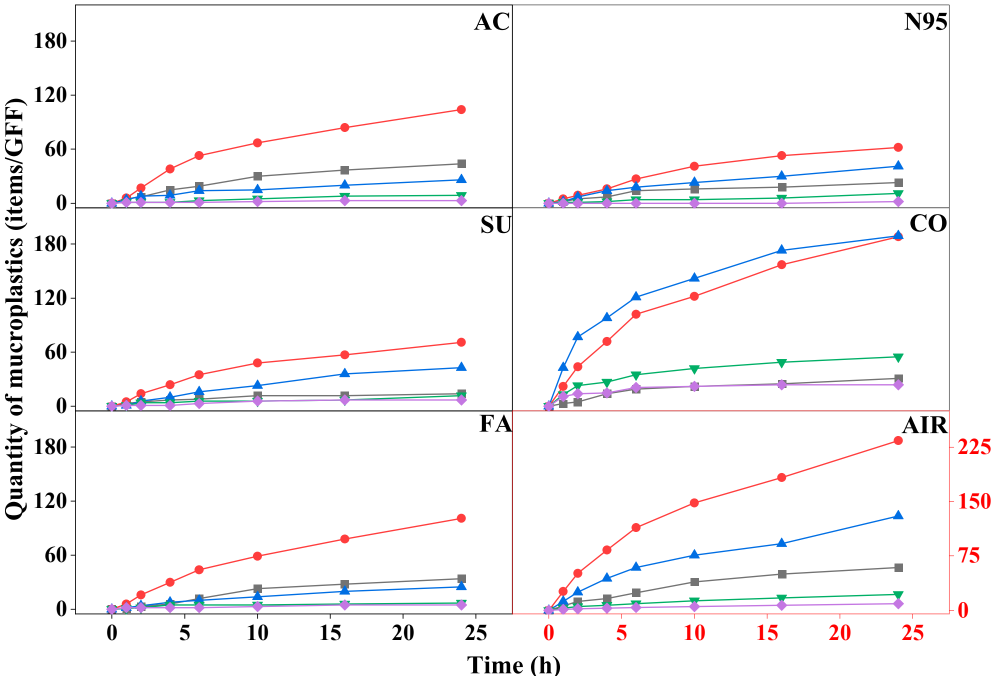

Figure S6. Six groups of microplastics collected from activated-carbon mask sample (AC), N95 mask sample (N95), surgical mask sample (SU), cotton mask sample (CO), fashion mask sample (FA) and blank sample (AIR) in indoor (top) and outdoor (bottom) conditions.

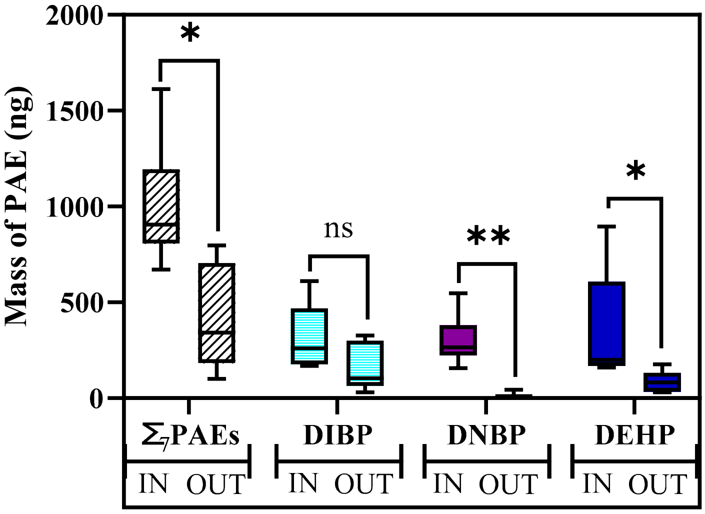

Figure S7. Mass of phthalates in GFFs from indoor (IN) and outdoor (OUT) conditions after 24 hours. ns: no significancy, * $p < 0.05$, ** $p < 0.01$.

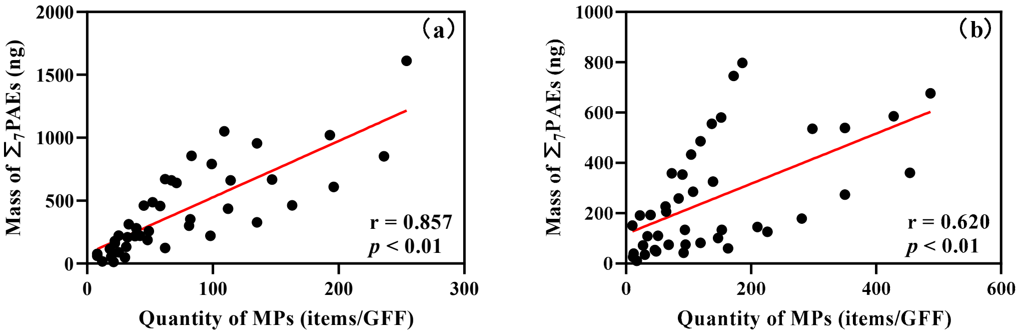

Figure S8. Linear correlation of MPs quantity and ∑7PAEs mass in (a) indoor and (b) outdoor conditions.

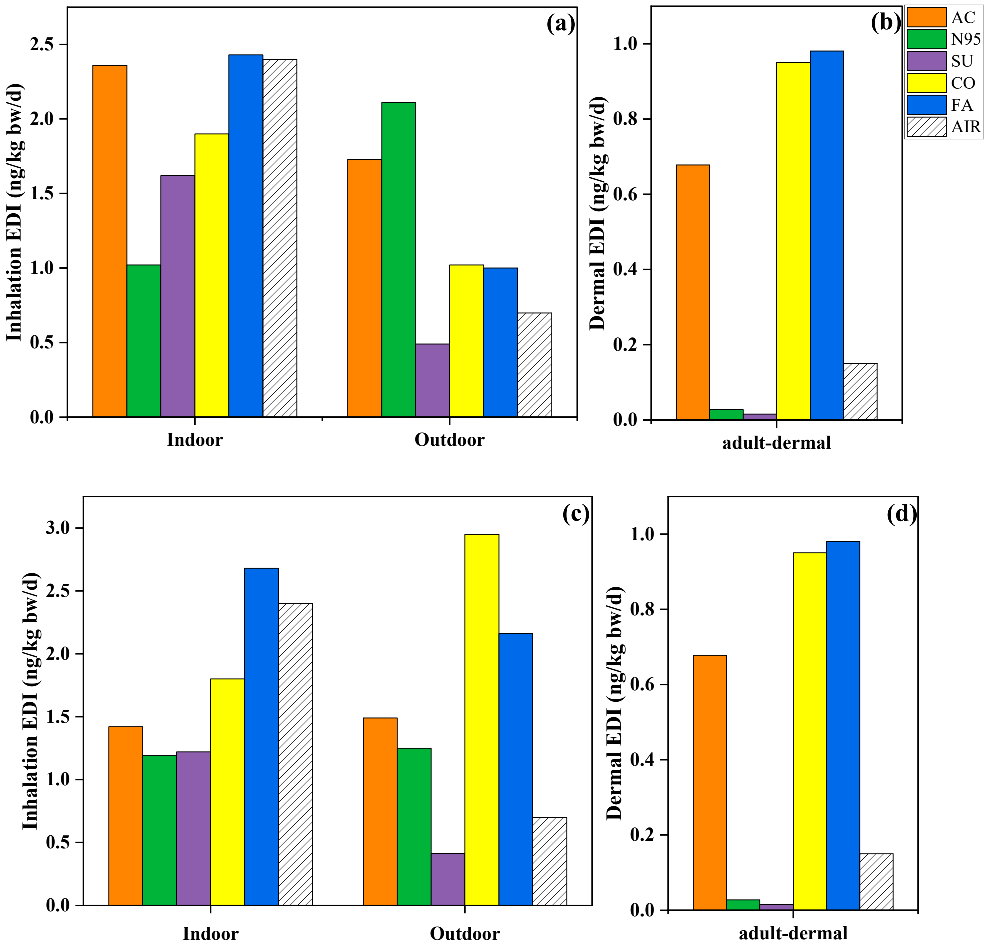

Figure S9. Inhalation (a,c) and dermal (b,d) estimated daily intake of phthalates of an adult while wearing activated-carbon mask (AC), N95 mask (N95), surgical mask (SU), cotton mask (CO), fashion mask (FA) and wearing no mask (AIR) in indoor and outdoor conditions. (a) and (b) simulated the condition that mask was changed after 4 hours and kept for later 2 hours, while (c) and (d) were based on the mass of phthalates and number of microplastics collected after 6 hours. Exposure pattern of children was the same as that of adults.
